# The Role of Age and Comorbidities in Esophagogastric Cancer Chemoradiation of the Frail Elderly (>70 Years): An Analysis from a Tertiary High Volume-Center

**DOI:** 10.3390/cancers15010106

**Published:** 2022-12-23

**Authors:** Philipp Linde, Markus Mallmann, Anne Adams, Simone Wegen, Jiaqi Fan, Johannes Rosenbrock, Maike Trommer, Simone Marnitz, Christian Baues, Eren Celik

**Affiliations:** 1 Department of Radiation Oncology, Cyberknife and Radiation Therapy, Faculty of Medicine and University Hospital of Cologne, University of Cologne, Kerpener St 62, 50937 Cologne, Germany; 2 Center for Integrated Oncology (CIO), University Hospital of Cologne, Faculty of Medicine and University of Cologne, Kerpener St 62, 50937 Cologne, Germany; 3 Institute of Medical Statistics and Computational Biology, Faculty of Medicine and University of Cologne, Kerpener St 62, 50937 Cologne, Germany

**Keywords:** elderly, esophageal cancer, frailty, chemoradiation, radiation toxicity, nutrition

## Abstract

**Simple Summary:**

Esophageal cancer (EC) is one of the most common cancers worldwide, with over 1.6 million new cases and 1.2 million deaths each year. There is insufficient data on management of elderly and frail EC patients. It is important to explore how elderly EC patients benefit from definite and neoadjuvant chemoradiation regimens. The choice of a schedule and the chemotherapy components in regard of age, comorbidities and several influencing risk factors remains challenging. In our retrospective analysis, combination of carboplatin and paclitaxel shows the benefits of preventing progressive disease and prolonging survival without increasing adverse reactions. Furthermore, trimodal treatment seems to be feasible and effective in the group of the elderly. We discuss current knowledge and the results of studies on the role of chemoradiation in frail elderly EC patients.

**Abstract:**

Elderly patients > 70 years of age with esophageal cancer (EC) represent a challenging group as frailty and comorbidities need to be considered. The aim of this retrospective study was to evaluate the efficacy and side effects of curative chemoradiation therapy (CRT) with regard to basic geriatric screening in elderly patients in order to elucidate prognostic factors. Thirty-four elderly patients > 70 years with EC treated at our cancer center between May 2014 and October 2018 fulfilled the selection criteria for this retrospective analysis. Treatment consisted of intravenous infusion of carboplatin/paclitaxel or fluorouracil (5-FU)/cisplatin with the intention of neoadjuvant or definite chemoradiation. Clinicopathological data including performance status (ECOG), (age-adjusted) Charlson comorbidity index (CCI), Frailty-scale by Fried, Mini Nutritional Assessment Short Form, body mass index, C-reactive protein to albumin ratio, and treatment-related toxicity (CTCAE) were assessed. Data were analyzed as predictors of overall survival (OS) and progression-free survival (PFS). All patients (ten female, 24 male) received combined CRT (22 patients in neoadjuvant, 12 patients in definite intent). Median age was 75 years and the ECOG index between 0 and 1 (52.9% vs. 35.3%); four patients were rated as ECOG 3 (11.8%). Median follow-up was 24 months. Tumors were mainly located in the lower esophagus or esophagogastric-junction with an T3 stage (n = 25; 75.8%) and N1 stage (n = 28; 90.3%). 15 patients (44.1%) had SCC, 19 patients (55.9%) AC. 26 of the patients (76.5%) were scored as prefrail and 50% were in risk for malnutrition (n = 17). In relation to the BMI, ten patients (29.4%) were ranked as overweight, and 15 patients were presented in a healthy state of weight (44.1%). Grade 3 acute toxicity (or higher) occured in nine cases (26.5%). Most of the patients did not show any late toxicities (66.7%). Trimodal therapy provides a significant prolonged OS (*p* = 0.049) regardless of age, but without impact on PFS. Our analysis suggests that chemoradiation therapy is feasible for elderly patients (>70 years) with tolerable toxicity. Trimodal therapy of EC shows a positive effect on OS and PFS. Further studies are needed to elucidate benefitting subgroups within the elderly. In addition to age, treatment decisions should be based on performance status, nutritional condition and multidisciplinary validated geriatric screening tools.

## 1. Introduction

Esophageal cancer (EC) is the second most common tumor of the gastrointestinal organ besides cancer of the stomach [1].Squamous cell carcinomas (SCC) of the esophagus are the most common histology in Asian countries, while in Western regions adenocarcinomas (Adeno) occur with increasing incidence [2,3]. Since life expectancy will also continue to increase over time, the number of elderly patients with EC is likely to rise in the future [4].

A person is often defined as “elderly” once that person is 65 years of age or older [5,6]. Elderly patients are often excluded from clinical trials or are at least underrepresented as a group [7,8,9]. Biological age as a prognostic factor is the focus of current discussions, but several studies have shown no prognostic impact of age [10,11].

Semrau et al. contrasted 152 patients with EC aged < 70 years treated with definite chemoradiation (dCRT; cisplatin/5-fluorouracil) with 51 patients aged ≥ 70 years [12]. Progression-free survival (PFS) showed a significant benefit in favor of the group ≥ 70 years, with no difference in overall survival (OS) in the two groups. In contrast, an analysis by Takeuchi et al. showed significantly lower efficacy and more severe toxicities (cisplatin/5-fluorouracil protocol) in selected elderly patients [13].

In the last decade, a landmark study by van Eyck et al. has significantly reduced chemotherapy-related toxicities using carboplatin/paclitaxel (Carb/Tax) [14]. The combination of Carb/Tax with simultaneous 41.4 Gy radiotherapy before surgery appears to be safe in the long term, even in patients over 75 years of age, and does not significantly increase the risk of toxicity-related deaths or postoperative mortality [15]. Based on their study, Haefner et al. stated that age and comorbidities should not be the main factors in the decision for dCRT or neoadjuvant chemoradiation therapy (nCRT) followed by surgical treatment [16]. The Charlson comorbidity index (CCI) ≤ 2 seems to be an (age-)independent prognostic factor associated with survival for elderly patients [17,18].

Due to the special characteristics of elderly patients, nomograms, geriatric assessment tools and established preoperative screening tools are applied to determine the performance status of the oldest patients prior to therapy decisions and initiation [19,20,21].

We here present a retrospective single-center analysis including trimodal therapy and definite CRT regimes in patients > 70 years with EC. We focus on acute and late toxicities, overall and progression free survival and try to elucidate the relevance of comorbidities in regard of therapy choice, and to identify explanatory variables.

## 2. Patients and Methods

### 2.1. Patient Cohort

We identified 34 patients aged > 70 years with EC (cT1-4, any N, any M) treated with either neoadjuvant or definitive CRT at our tertiary cancer care centre between May 2014 and October 2018. Due to the time period covered by this study and changing clinical practice, chemotherapy regimens and radiation techniques vary. Inclusion criteria were age > 70 years, newly diagnosed histologically proven AC or SCC of the esophagus and definite or neoadjuvant CRT using conventional (anterior-posterior (APPA)fields, 3D-conformal multi-field or intensity-modulated radiotherapy (IMRT)) techniques. We excluded patients who were treated for recurrent disease, who had previously received CRT or had received definitive treatment without chemotherapy. A systematic audit of patients’ charts and reports was performed to identify patient and treatment characteristics, reported acute and late toxicities, and treatment-related outcomes.

### 2.2. Radiotherapy

All patients were simulated in supine position in a universal/wing board for mid to distal esophageal tumors or immobilized using a customized long thermoplastic mask for upper EC. Once contraindications (e.g., risk of aspiration, allergy, renal insufficiency) had been discounted, the contrast medium was administered in a standardized manner both intravenously and orally. The gross tumor volume (GTV) was identified on the pre-chemotherapy extent of the disease using the endoscopy report and initial positron emission tomography in combination with a computed tomography (PET/) CT scan. The entire esophageal wall, including any disease extending through the wall, was contoured as GTV, as were any (PET/) CT-avid or pathologically enlarged lymph nodes. The clinical target volume (CTV) encompassed mediastinal lymph nodes, the peri-esophageal lymph nodes, and the submucosal spread longitudinally along the esophagus. This required a 1.0–1.5 cm radial expansion on the GTV and a 3–4 cm expansion superiorly and inferiorly. The planning target volume (PTV) was uniformly generated with the addition of 0.7 cm.

Radiotherapy was provided once a day, five times a week, except weekends and holidays, with a daily dose of 1.8 Gy. The total doses administered on the PTV were 50.4 Gy and a subsequent boost of 9 Gy on the GTV for dCRT, and 41.4 Gy for nCRT.

### 2.3. Chemotherapy

Patients received chemotherapy according to a treatment plan based on ECOG performance status, comorbidity, and the presence of specific contraindications to the planned agents. All patient cases were discussed in a multidisciplinary tumor board (both pre and post) and the concomitant chemotherapy was finally prescribed by the treating radiation oncologist. Intravenous chemotherapy consisted of either two courses of cisplatin/5-fluorouracil (cisplatin [75 mg/m^2^ body-surface area] on the first day combined with 5-fluorouracil [1000 mg/m^2^] continuous infusion daily for four days) or cycles of carboplatin/paclitaxel (Carb/TAX; carboplatin [AUC 2 mg/mL per min] combined with paclitaxel [50 mg/m^2^ body-surface area], weekly). Patients who were to receive taxane-based dCRT were planned for five to six administrations; four applications were targeted in the neoadjuvant setting.

Treatment with Carb/TAX was given alongside premedication to avoid hypersensitivity reactions (dexamethasone, dimetinden maleate and H2 antagonists). The platinum-containing regimen was applicated after adequate i.v. prehydration, mannitol and i.v. antiemetics (5HT3 antagonists and dexamethasone), followed by i.v. posthydration. If necessary, additional antiemetics (5HT3 antagonists, corticosteroids, dimenhydrinate and metoclopramide) were used to treat prolonged nausea after other causes of nausea had been ruled out (e.g., hyponatremia). A complete blood count and serum chemistry test, including creatinine clearance, were performed at least once a week.

Dose reduction or de-escalation of chemotherapy was considered if grade 3 to 4 hematological toxicity emerged. If severe radiation-related toxicity occurred, a patient-centred decision on dose reduction or discontinuation of chemotherapy was considered.

### 2.4. Monitoring under Chemoradiation and Associated Supportive Measures

Treatment toxicity was assessed daily during chemotherapy and weekly during radiotherapy. If clinically indicated, toxicity was monitored more frequently. The documentation was standardised (Common Terminology Criteria for Adverse Events (CTCAE)). Supportive care included symptom management, e.g., nutritional counselling, enteral or parenteral nutrition, pain anamnesis and therapy according to the WHO standard, and supportive hospitalisation if needed.

### 2.5. Aftercare

All patients were offered structured follow-up in our department in addition to the primary medical-oncological follow-up. It took place for the first time 8–10 weeks after the end of treatment and then at intervals of three to six months during the first year. The follow-up included a medical history, an orienting clinical examination, and anamnesis in accordance with the LENT/SOMA criteria [22]. Chest-abdomen-pelvis CT scans for staging and evaluation of the former radiation region and an endoscopy ordered and provided by the primary care provider (usually an oncologist) were reviewed, and in individual cases, scheduled for the patient. In the second year of follow-up, patients were regularly visited at least every 6–12 months until progression of disease or death. Patients and their primary care physicians were encouraged to report complications occurring after chemoradiation immediately to our department. If a scheduled follow-up appointment was missed, patients were contacted (by phone or by letter) by our patient management. Relapse of disease was documented by histological biopsy when possible. In case of missing information, the database of the University Hospital of Cologne was checked for information on the current status of the patient.

### 2.6. Evidence-Based Definitions, Statistical Analysis, and Ethical Considerations

Acute and late toxicity was scored retrospectively according to CTCAE v5.0 [23]. Acute toxicities were defined as occurring during radiotherapy and 90 days after the start of radiotherapy; from day 91 onwards they are late side effects. Performance status was scored according to the ECOG index [24]. The (age adjusted) Charlson comorbidity index was used to analyze the comorbidity burden of our cohort [25,26]. Patients were assessed using the Frailty scale [27]. With this criterion, in many clinical situations patients have a less favorable course of treatment and disease [28]. It is used for integrated assessment and in shared decision making. Nutritional status and body composition were determined using the short-form Mini Nutritional Assessment (MNA-SF) scale and body mass index (BMI) [29,30]. To independently assess the prognosis of EC patients, serum C-reactive protein (CRP) and albumin concentrations were collected, and the CRP/albumin ratio (CAR) was calculated [31,32,33]. The standard values were used on the basis of the reference standard of the central laboratory of the University Hospital of Cologne: CRP < 3 μg/L; albumin 35–52 g/L). Categorization was set CAR > 0.5 vs. <0.5. Here, we were interested in the fact that patients with high CAR usually have severe tumor-related inflammatory responses or are in poor nutritional status and therefore may benefit from anti-inflammatory therapy and nutritional support [34,35].

Survival data were calculated according to the Kaplan-Meier method [36]. OS was defined as the interval from the first day of treatment to death or to the last follow-up time point still alive. PFS was calculated from the first day of treatment until death or diagnosis of relapse (local or distant metastases) or last follow-up alive. Univariate analyses were performed using log rank testing and Cox regression [37]. A *p*-value of <0.05 was defined as statistically significant. Calculations and data management were performed with SPSS^®^-statistics software v.28.0.1.0.

The study was conducted in accordance with the Declaration of Helsinki in its latest version. All patients gave written informed consent before the start of treatment. Due to the retrospective nature, from the point of view of the local ethics committee, there is no professional consultation obligation for the North Rhine physicians according to § 15 para. 1 of the professional code of conduct.

## 3. Results

Median follow-up for the entire cohort was 24 months. Radiotherapy was completed by all of the patients and all patients received initially prescribed dose (Gy).

Median age of the 34 patients in our cohort at the start of therapy was 75 years (range 71–82), ten patients were female, 24 were male. 15 patients (44.1%) had SCC, 19 patients (55.9%) AC. The majority presented with an ECOG index between 0 and 1 (52.9% vs. 35.3%), T3 stage (n = 25; 75.8%) and N1 stage (n = 28; 90.3%). The majority of patients were treated with nCRT (n = 22; 64.7%) and received surgery. Myocardial infarction, congestive heart failure, peripheral vascular disease, chronic obstructive pulmonary disease, and uncomplicated diabetes were the most common comorbidities and therefore included in the Charlson index scores. 26 of the patients (76.5%) were scored as prefrail and 50% were in risk for malnutrition (n = 17). In relation to the BMI, ten patients (29.4%) were ranked as overweight and 15 patients were presented in a healthy state of weight (44.1%).

For detailed patient, scoring and treatment characteristics see Table 1 and Table 2.

### 3.1. Chemotherapy

29 of our 34 patients received concurrent Carb/Tax chemotherapy, one was treated with cisplatin/5-fluorouracil, two patients received Tax monotherapy. Two patients did not receive any chemotherapy based on the interdisciplinary assessment of the treatment team.

### 3.2. Toxicities

For detailed analysis of acute and late toxicity see Table 3 and Table 4.

#### 3.2.1. Acute

Grade 3 acute toxicity (or higher) was found in nine cases (26.5%). Generally, maximum acute toxicity during or after CRT was grade 0 to 1 in 12 patients (35.3%), grade 2 in 13 patients (38.2%), grade 3 in seven (20.6%), grade 4 in one (2.9%), and grade 5 in one patient (2.9%); mainly hematotoxicity, (odyno-)dysphagia, nausea, loss of appetite, and fatigue. The patients with grade 5 toxicity presented as follows: 80 years, female, ECOG 3, CCI 2, cT3, SCC, and dCRT (Tax mono). Grade 5 toxicity was related to hematotoxicity.

#### 3.2.2. Late

Most of the patients did not show any late toxicities (66.7%). Grade 3 late toxicity (or higher) was found in two of 30 evaluable cases. Maximum late toxicity after CRT was grade 1 in seven patients (23.3%), grade 2 in one (3.3%), grade 3 in one (3.3%), grade 4 in one (3.3%), and grad 5 in one (3.3%); solely (odyno-)dysphagia. The patient with grade 5 toxicity presented as follows: 67 years, male, ECOG 2, CCI 5, cT3, SCC, and dCRT (Carb/TAX). Grade 5 toxicity was related to fistula.

### 3.3. Survival Analysis

**Figure 1:**
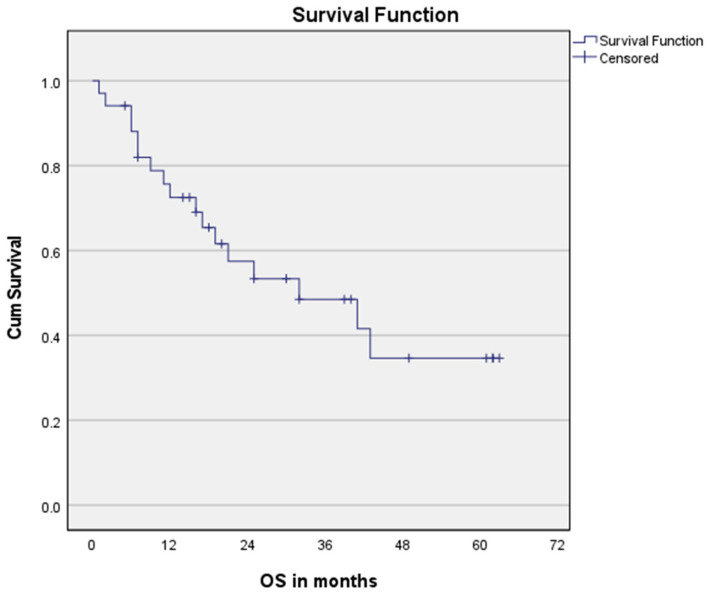
Overall survival for the entire cohort. n = 34.

**Figure 2:**
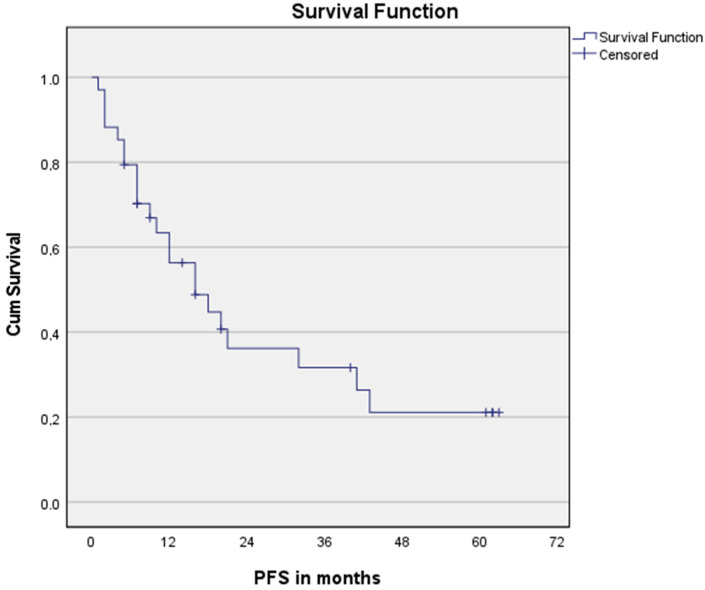
Progression-free survival for the entire cohort. n = 34.

The median OS of all patients > 70 years was 32 months and the median PFS was 16 months, see Figure 1 and Figure 2, respectively. The median OS for SCC patients was 43 months vs. 21 months for AC (*p* = 0.008).

**Figure 3:**
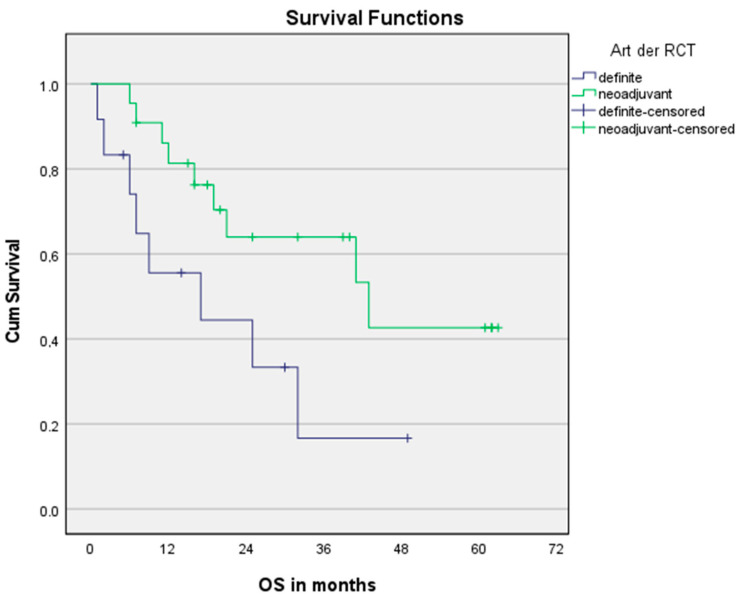
Overall survival for nCRT and dCRT. n = 34. Log Rank (Mantel-Cox): *p* = 0.049.

**Figure 4:**
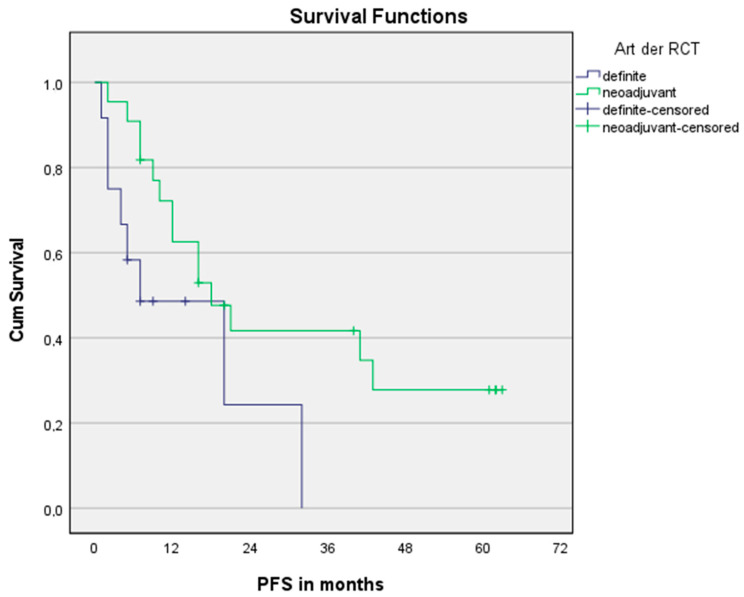
Progression-free survival for nCRT and dCRT. n = 34. Log Rank (Mantel-Cox): *p* = 0.057.

In the univariate Cox regression model, treatment regimen (nCRT vs. dCRT) significantly affected OS, but not PFS, see Figure 3 and Figure 4, respectively. The trend shown is independent of age. Pre-therapeutic ECOG PF of 0–1 was one parameter significantly affecting OS and PFS, too. An elevated CAR > 0.5 seems to be a predictor of PFS without being statistically significant.

Detailed results of univariate Cox regression analysis are demonstrated in Table 5.

## 4. Discussion

This study assessed the OS, PFS and treatment-related toxicities of patients older than 70 years with EC treated with nCRT and dCRT in relation to comorbidities and common baseline performance scores. Biological age has been addressed as a prognostic parameter in previous RT studies on EC [38,39]. With the increase in average life expectancy, the number of elderly EC patients is steadily increasing year by year [40,41].

In our small but representative cohort of patients over 70 years, a significant advantage in terms of OS and PFS was seen in the nCRT group and concomitant Carb/TAX chemotherapy was well-tolerated in both treatment groups. Herskovic et al. were able to remarkably demonstrate the therapeutic benefit of concurrent chemotherapy in definite treatment using radiotherapy [42]. Ji et al. were able to show a clear improvement in efficacy with the 5-FU prodrug Tegafur in a cohort of elderly patients aged 70 to 85 years who were randomized to RT alone and simultaneous dCRT [43]. High-grade toxicities (grade 3) with leukopenia occurred in the combination arm (9.5% vs. 2.7%). Two-year overall survival (OS) was significantly improved (*p* = 0.002) demonstrating the feasibility and tolerability with higher efficacy, substantially improving treatment options for elderly patients [44,45,46].

Surgical treatment of EC is increasingly accepted for elderly people defined as aged over 70 years. Not differing outcome and long-term results in the selected elderly from those reported for younger patients offer the possibility of risk/opportunity stratification and assessment [47,48,49,50]. Here, based on a comparison of the CROSS trial with historical controls (e.g., RTOG 8501 and Intergroup [INT] 0123) and retrospective studies, radiosensitizing with Carb/TAX seems more tolerable than cisplatin/5-fluorouracil [51,52,53].

The retrospective comparisons by Munch et al. between the two established chemotherapy regimens (cisplatin/5-fluorouracil or Carb/TAX) are in line and highlight the results concerning the moderate acute and late toxicities due to chemotherapy in our cohort [54,55]. 

Our data confirm that risk factors in the group of elderly should never be considered in absolute order to avoid selection bias. Special attention should be given to the fact that the “advanced age” factor is associated with lower referral of older patients to specialists, increased administration of suboptimal therapies and increased rejection of therapy by the patients themselves [56,57,58,59].

In addition to the experienced clinician’s view, validated screening and geriatric assessment tools are needed in order to achieve an objective patient selection [60,61,62,63]. Screening tools such as O-POSSUM and predictive models such as those published by Steyerberg et al. and the International Esodata Study Group should generally be considered when stratifying elderly patients for more intensive or less intensive therapy [64,65,66,67,68]. More recent data highlights the critical role of rigorous supportive care [69]. For example, integrating prophylactic intravenous hydration, nutritional counselling and more frequent treatment visits towards the end of treatment could reduce emergency department visits and treatment interruptions in this vulnerable population of the elderly. Recently, prehabilitation has emerged as a way to prepare a patient for any major surgery [70,71]. If patients can be prevented from worsening their physical and nutritional condition, this could have a significant impact on overall cancer treatment [72,73].

Of course, the patient’s wish for therapy, quality of life (QOL) scoring, and the involvement of his or her relatives should also be emphasized here [74,75]. Specific radiooncological screening, prehabilitation, and assessment tools are rare; here, interdisciplinary exchange and the development of valid instruments are to be wished and must set as a goal for the future [76,77].

We observed that ECOG performance status (0–1) is an age-independent predictor of survival. Recently, Hulshof et al. showed within their prospective ARTDECO trial that escalating the radiation dose (DE) does not improve local tumor control or survival [78]. Interestingly, the majority of patients were elderly (mean 71 years) and mainly had an ECOG-PS of 0 or 1. The results may therefore not be applicable to younger patients (around 50 years of age), who may represent a beneficial subgroup of a DE. Consistent with our retrospective data, there were no significant differences in overall toxicities between the 1:1 randomized arms of elderly patients (DE vs. standard dose). With modern staging and (pre-)treatment approaches, similar outcomes appear to be achievable for the elderly as in younger cohorts with EC. [16,79,80,81]. Esophagectomy in the elderly is also less used and is associated with a potential increase in peri- and postoperative complications but is performed in selected patients with acceptable outcomes [82,83,84,85,86]. Minimally invasive esophagectomy may minimize mortality in elderly patients [87]. Vlacich et al. presented a review of the National Cancer Database [45]. Patients ≥ 80 years were more likely to receive no treatment in the non-definite setting and less likely to receive trimodal therapy and esophagectomy alone in the definite setting. Considering individual comorbidities and the known pharmacokinetic properties and modes of action in the elderly, they should be treated as younger patients [88,89]. Adjuvant immunotherapy, for example, should complement the curative treatment approach in the case of pathologically incomplete response after trimodal therapy, even in older patients [41,90]. The median age in the CheckMate 577 registration trial was 62 years (PD-1 inhibitor arm) vs. 61 years (placebo arm) in an ECOG of 0–1, too and well tolerated.

## 5. Limitations

Our retrospective analysis comes with limitations. Treatment compliance is one of the most important endpoints in the treatment of frail elderly patients. All patients who started chemoradiation with curative intent should be included and e.g., shown within a CONSORT flow chart. As this is a retrospective cohort study, the design may be prone to sampling bias, which we must cite as our major limitation. The results should therefore be interpreted with caution.

## 6. Conclusions

The data support the supposition that chemoradiation therapy is feasible for the elderly patient group (>70 years) with tolerable toxicities. Trimodal therapy shows a positive effect on OS and PFS. Further studies are needed to elucidate benefitting subgroups within the elderly. Treatment decisions should no longer be based solely on chronological age, but should include performance status, nutritional condition and validated geriatric screening tools. With modern treatment approaches and well-tolerated cytostatic agents, similar results seem to be achievable as in younger patients.

## Figures and Tables

**Table 1:** Patient and treatment characteristics (n).

**Sex**		**Grading**	
Female	10 (29.4%)	1	1 (2.9%)
Male	24 (70.6%)	2	16 (47.0%)
**Age**		3	11 (32.3%)
Median	75 y	Gx/Unknown	6 (17.6%)
Mean	75.4 y	**T-stage**	
Range	71–82 y	2	7 (20.5%)
**ECOG**		3	25 (73.5%)
0	18 (52.9%)	4	1 (2.9%)
1	12 (35.3%)	Tx/Unknown	1 (2.9%)
3	4 (11.8%)	**N-stage**	
**Tumor site**		0	1 (2.9%)
Cervical	1 (2.9%)	1	28 (82.3%)
Upper thoracic	2 (5.9%)	2	1 (2.9%)
Middle thoracic	11 (32.4%)	3	1 (2.9%)
Lower thoracic	20 (58.8)	Nx/Unknown	3 (8.8%)
**Histology**		**M-stage**	
Adeno	19 (55.9%)	0	31 (91.1%)
SCC	15 (44.1%)	1	2 (5.8%)
		Mx/Unknown	1 (2.9%)
**AJCC/UICC stage ^I^**		**SCC**	**Adeno**
II		1 (2.9%)	None
III		12 (35.3%)	13 (38.2%)
IVA		2 (5.8%)	None
IVB		None	2 (5.8%)
Unknown		None	4 (11.8%)
**Chemotherapy**		**RT technique**	
Neoadjuvant	22 (64.7%)	APPA (two-field)	6 (17.6%)
Definite	12 (35.2%)	3D-CRT (multi-field)	17 (50.0%)
**Agents**		IMRT	10 (29.4%)
Carb/TAX	29 (85.2%)	Unknown	1 (2.9%)
Others	3 (8.8%)		
None	2 (5.8%)		

Adeno adenocarcinoma, APPA anterior/posterior fields, Carb/TAX Carboplatin and paclitaxel, ECOG Eastern Cooperative Oncology Group, IMRT intensity modulated radiation therapy, RT radiation therapy, SCC squamous cell carcinoma, 3D-CRT three-dimensional conformal radiation therapy, Unknown Due to the retrospective character of the study, y years. ^I^ 8th edition, American Joint Committee on Cancer (AJCC)/Union for International Cancer Control (UICC) staging of cancers of the esophagus and esophagogastric junction.

**Table 2:** (Geriatric and categorical) Screening score characteristics (n).

**Charlson Score ^I^**		**SCC**	**Adeno**
≤3		6 (17.6%)	5 (14.7%)
≥3		9 (26.4%)	14 (41.1%)
**Age adjusted Charlson score**		**Active smoking**	
≥5	34 (100%)	Yes	12 (35.2%), (5 SCC (14.7%))
**Frail scale**		**Mini Nutritional Assessment Short Form**	
Median	1 pt	Median	8.5 pt
Mean	1.2 pt	Mean	8.1 pt
Range	0–3 pt	Range	3–12 pt
Fit and agile (0 pt)	7 (20.6%)	Normal nutritional state (12–14 pt)	2 (5.9%)
Prefrail (1–2 pt)	26 (76.5%)	Risk for malnutrition (8–11 pt)	17 (50%)
Frail (≥3 pt)	1 (2.9%)	Malnutrition (0–7 pt)	15 (44.1%)
**BMI (weight (kg)/[height(m)]^2^)**		**Weight (kg)**	
Median	24.6	Median	76
Mean	24.5	Mean	75.1
Range	15.2–37.2	Range	35–120
Obesity (30 pt or higher)	4 (11.8%)	**C-reactive/albumin ratio**	
Overweight (25.0 to 29.9 pt)	10 (29.4%)	Median	0.1
Healthy Weight (18.5 to 24.9 pt)	15 (44.1%)	Mean	0.5
Underweight (below 18.5 pt)	5 (14.7%)	Range	0.1–7.0

Adeno adenocarcinoma, BMI Body mass index, pt points, SCC squamous cell carcinoma, y years. ^I^ The final score was calculated for each patient by considering all comorbid conditions present with the inclusion of EC as a cancerous disease (+two points).

**Table 3:** Acute treatment related toxicities according to Common Terminology Criteria for Adverse Events (CTCAE, v5.0), absolute number of patients (n = 34).

CTC Grade	0	%	1	%	2	%	3	%	4	%	5	%
Hematological	7	20.6	10	29.4	13	38.2	2	5.9	1	2.9	1	2.9
Odynodysphagia	19	55.9	10	29.4	2	5.9	3	8.8	0	0	0	0
Nausea	28	82.4	6	17.6	0	0	0	0	0	0	0	0
Skin toxicity	22	64.7	11	32.4	0	0	1	2.9	0	0	0	0
Fatigue	24	70.6	7	20.6	2	5.9	1	2.9	0	0	0	0
Cardiopulmonary	34	100.0	0	0	0	0	0	0	0	0	0	0
Inappetence/loss of weight	32	94.1	1	2.9	0	0	1	2.9	0	0	0	0
Other ^1^	30	88.2	3	8.8	0	0	1	2.9	0	0	0	0

^1^ Other: diarrhea, thrush esophagitis, reflux esophagitis.

**Table 4:** Late treatment related toxicities according to CTCAE v5.0, absolute number of patients.

	All Grades		Grade 1–2		Grade 3–5	
	n	%	n	%	n	%
Odynodysphagia	30	100	0	0	3	9.9
Nausea	30	100	3	10.0	0	0
Skin toxicity	30	100	4	13.3	0	0
Fatigue	30	100	1	3.3	0	0
Cardiopulmonary	30	100	1	3.3	0	0
Inappetence/loss of weight	30	100	1	3.3	0	0
Other ^1^	30	100	3	10.0	0	0

^1^ Other: cardiac decompensation, renal failure, N-STEMI, progression of Parkinson’s disease.

**Table 5:** Univariate Cox regression and subgroup analysis.

Parameter	OS HR [95% CI]	*p*-Value	PFS HR [95% CI]	*p*-Value
**Treatment regimen** **(nCRT vs. dCRT)**	0.381 [0.146; 0.998]	**0.049**	0.411 [0.164; 1.028]	0.057
**Age**				
70–74 y (nCRT vs. dCRT)	0.067 [0.007; 0.674]	**0.022**	0.233 [0.050; 1.077]	0.062
**ECOG (2–3 vs. 0–1)**	17.808 [3.903; 81.258]	**<0.001**	11.391 [3.079; 42.138]	**<0.001**
**BMI**				
Healthy Weight (nCRT vs. dCRT)	0.159 [0.029; 0.878]	**0.035**	0.500 [0.140; 1.789]	0.286
**T-stage**				
T3 (nCRT vs. dCRT)	0.095 [0.022; 0.407]	**0.002**	0.205 [0.062; 0.678]	**0.009**
**Grading**				
G3 (nCRT vs. dCRT)	0.309 [0.072; 1.326]	0.114	0.233 [0.053; 1.033]	0.055
**Histology**				
SCC (nCRT vs. dCRT)	0.161 [0.018; 1.445]	0.103	0.142 [0.016; 1.290]	0.083
AC (nCRT vs. dCRT)	0.284 [0.073; 1.112]	**0.071**	0.469 [0.128; 1.713]	0.252

dCRT definite chemoradiation therapy, HR Hazard ratio, CI Confidence interval, nCRT neoadjuvant chemoradiation therapy, OS Overall survival, PFS Progression-free survival, y years.

## Data Availability

The data are included in the article. Further details can be obtained from the corresponding author via e-mail.

## Abbreviations

3D-CRT	3-dimensional conformal radiotherapy
AC	Adenocarcinoma
Carb/TAX	Carboplatin and paclitaxel
dCRT	Definite chemoradiation therapy
EC	Esophageal cancer
GTV	Gross target volume
Gy	Gray
IMRT	Intensity-modulated radiotherapy
nCRT	Neoadjuvant chemoradiation therapy
OS	Overall survival
PET/CT	Positron emission tomography/computed tomography
PFS	Progression-free survival
PTV	Planning target volume
SCC	Squamous cell carcinoma
QOL	Quality of life
